# Protective effect of interleukin-36 receptor antagonist on liver injury induced by concanavalin A in mice

**DOI:** 10.22038/ijbms.2020.35614.8492

**Published:** 2020-05

**Authors:** Xiao Peng, Xiuhe Pan, Jun Tan, Yan Li, Mingcai Li

**Affiliations:** 1 Department of Immunology, Medical School of Ningbo University, Ningbo 315211, China; 2 Department of Hepatology, HwaMei Hospital, University of Chinese Academy of Sciences, Ningbo 315010, China

**Keywords:** Concanavalin A (ConA), Hydrodynamic-based gene – delivery, IL-36Ra, Inflammation, Liver injury, Plasmid

## Abstract

**Objective(s):**

Interleukin-36 receptor antagonist (IL-36Ra) is a new member of the IL-1 family that exhibits anti-inflammatory activity in a variety of inflammatory and immune diseases. Our purpose was to determine the effect of IL-36Ra on liver injury in a mouse hepatitis model induced by concanavalin A (ConA).

**Materials and Methods:**

Mice were treated with IL-36Ra DNA or pcDNA3.1 control plasmid using a hydrodynamic gene delivery approach.

**Results:**

Our data reveal that treatment with IL-36Ra decreased liver inflammation and serum level of aminotransferases. Furthermore, IL-36Ra reduced ConA-induced pro-inflammatory cytokines (interferon-γ, tumor necrosis factor-α, and IL-17A) production when compared to control plasmid.

**Conclusion:**

Our results demonstrated that IL-36Ra is a critical protector against ConA-induced liver injury.

## Introduction

Interleukin-1 (IL-1) family of cytokines comprises 11 members, namely IL-1α, IL-1β, IL-1 receptor antagonist (IL-1Ra), IL-18, IL-36Ra, IL-36α, IL-37, IL-36β, IL-36γ, IL-38, and IL-33, respectively. IL-36Ra is the fifth newly discovered cytokine of the IL-1 family, which binds to IL-1 receptor-related protein 2 (IL-1Rrp2), also known as IL-1R6 signaling antagonist (1). It was identified based on its sequence homology with other IL-1 family members and was formerly known as IL-1 family member 5 (IL-1F5) (2). The IL-36Ra protein lacks a signal peptide and shares 52% amino acid homology with IL-1Ra (3). The human IL-36Ra open reading frame encodes a protein containing 155 amino acids, which is constitutively expressed by keratinocytes (4). IL-36Ra mRNA has been found to be ubiquitously present in the pathogenesis of many diseases, including general pustular psoriasis (5), neutrophilic airway inflammation (6), brain tissue inflammation (7), alopecia areata (8), and arthritis (9).

IL-36 cytokines that are members of the IL-1 superfamily include IL-36α, IL-36β, IL-36γ, and IL-36Ra. Interestingly, IL-36α, IL-36β, and IL-36γ have pro-inflammatory effects, while in contrast IL-36Ra exerts anti-inflammatory activities (10). IL-36α, IL-36β and IL-36γ activate nuclear factor-kappa B (NF-κB) through IL-1Rrp2/IL-1R6, and IL-1 receptor accessory protein (IL-1RAcP) (11, 12). Furthermore, IL-36α, IL-36β and IL-36γ can activate NF-κB and mitogen-activated protein kinases (MAPKs) and result in inflammatory cytokines secretion. In contrast, IL-36Ra inhibits the effect of IL-36 cytokines on NF-κB and MAPKs activation (11). IL-1Rrp2/IL-1R6 agonists enhance the capability of CD4^+^T cells to produce and secrete interferon (IFN)-γ and IL-17A in a dose-dependent manner (13). 

Hepatitis is one of the most common diseases, which threatens human health and has a significant morbidity and mortality. Importantly, one of the most common causes of liver injury is autoimmune hepatitis (14). Concanavalin A (ConA)-induced hepatitis is a well-established murine model for human acute hepatitis that mimics human T-cell-mediated hepatitis condition in many respects (15), including markedly increased serum levels of aminotransaminase, pathological changes, and pro-inflammatory cytokine overproduction (16, 17). Scheiermann *et al.* (18) recently reported that IL-36Ra can weaken the expression of the C-C chemokine CCL20 and impair recovery in the late phase of murine acetaminophen-induced liver injury. However, the role of IL-36Ra in ConA-induced liver injury is still unclear.

In this study, we investigated the significance of IL-36Ra in T cell-mediated hepatitis through a ConA-induced mouse hepatitis model. 

## Materials and Methods

***Experimental animals***

Male pathogen-free, BALB/c mice (6–8-weeks-old), weight ranging from 18 to 22 g, were obtained from Zhejiang University School of Medicine (Hangzhou, China). All experimental mice in this study were maintained and housed at the Animal Experimental Center of Ningbo University under standard laboratory conditions. Mice were provided standard water and food *ad libitum* in this study. The mice were allowed to adapt to the new environment for 5 days before the experiment, which was under a protocol approved by the Experimental Animal Ethics Committee of Ningbo University (SYDW20151001001).

***Hydrodynamic plasmid injection***

Plasmid DNA for IL-36Ra (constructed by our laboratory) was obtained from *Escherichia coli* using Plasmid Extraction kit (Tiangen Biotech, Beijing, China), and the plasmid DNA was dissolved in saline at a concentration of 50 μg/ml. Plasmid DNA was introduced into mice at a dose of 5 mg/kg using a hydrodynamic-based gene transfer approach (19) 24 hr before ConA (Sigma, St. Louis, MO., USA) injection. In our experiment, 1.2 mg plasmid DNA was diluted in 24 ml of saline for 12 mice (50 μg/ml) injection dosage. Plasmid pcDNA3.1-IL-36Ra-V5-His was injected into mice via the tail vein within 3 to 8 sec. The mice typically recovered from the injection within 5 to 10 min.

***Detection of exogenous IL-36Ra in mouse liver***

Livers were harvested from mice for studying IL-36Ra expression by reverse transcription-polymerase chain reaction (RT-PCR) and western blot. Mice were euthanized at 8, 24, 48, and 72 hr after the plasmid injection; each specified time point includes four mice. Another four mice were treated with an equal amount of saline and euthanized at 0 hr, as the normal control group.

For RT-PCR analysis, the total RNA was extracted from livers using RNAiso Plus Reagents (TaKaRa, Dalian, China) according to the manufacturer’s instructions. The RNA was then used to synthesize the cDNA with a HiFiScript cDNA First Strand Synthesis Kit (CWBIO, Beijing, China). The PCR was performed with 2×EasyTaq PCR SuperMix, and the IL-36Ra forward and reverse primer sequences: 5′-CGGGGTACCATGGTCCTGAGTGGGGC-3′ and 5′- GCTCTAGAGTCACACTGCTGGAAGTAGAAGTC-3′ were used for detecting IL-36Ra mRNA. The PCR conditions were as follows: 30 sec at 94 ^°^C for denaturation; 40 sec at 59 ^°^C for annealing; 70 sec at 72 ^°^C for extension; and 30 cycles. The PCR reaction was also performed with the housekeeping gene β-actin for normalization of results. The primer sequences for β-actin were 5’-TCCTGTGGGCATCCATGAAACT-3’; and 5’-GAAGCACTTGCGGTGCACGAT-3’. Western blot was used to detect IL-36Ra protein expression in the liver. Proteins were extracted from liver tissues using a RIPA Lysis Buffer (BeyoTime, Beijng, China), according to the manufacturer′s instructions. The proteins from representative mice were then subjected to western blot analysis. Membranes were incubated with 1:800 diluted anti-His-tag mouse primary antibodies (BeyoTime, Beijng, China) at 4 ^°^C for 14 hr. The membranes were further washed with Tris-buffered saline Tween 20 (TBST) buffer three times, and then incubated with 1:5000 diluted horseradish peroxidase (HRP)-conjugated goat anti-mouse IgG secondary antibodies for 45 min. Then, the protein bands were visualized by the enhanced chemiluminescence reaction method (Amersham Pharmacia Biotech, Piscataway, NJ, USA). 

***Treatment groups***

Forty-eight mice were randomly divided into four groups, which include IL-36Ra-treated group (IL-36Ra/ConA), pcDNA3.1-treated group (pcDNA3.1/ConA), saline-treated group (saline/ConA), and normal control group (saline only), with twelve mice included in each study group. The mice in the IL-36Ra-treated group and pcDNA3.1-treated group were intravenously injected with 2 ml of pcDNA3.1-IL-36Ra-V5-His (50 μg/ml) and 2 ml of pcDNA3.1 (50 μg/ml), respectively, while the saline-treated group and normal control group were injected with the same volume of saline. After 24 hr, the mice in groups (IL-36Ra/ConA, pcDNA3.1/ConA, and saline/ConA) were challenged with 240 μl of ConA at a dose of 15 μg/g via tail vein injection (20), while the mice in the normal control group were administered an equal volume of saline. Blood samples were collected by cardiac puncture at 8 hr after ConA injection, stored at room temperature for 30 min, followed by centrifugation at 3,000 rpm at 4°C for 10 min, and then frozen at -40°C until further analysis. Initial study found that the distribution of ConA was specific for the liver (21). Therefore, mouse livers were harvested from euthanized mice at 8 and 24 hr after ConA administration for histopathological assay and analysis of cytokine mRNA expression.

***Assay of serum aminotransaminase activity***

Alanine aminotransferase (ALT) and aspartate aminotransferase (AST) activities in mouse serum were measured by standard photometric methods using an ALT and AST diagnostic reagent kit from Nanjing Jiancheng Bioengineering Institute (Nanjing, China).

***Mouse liver histopathological examination***

Mouse livers harvested at 8 hr and 24 hr were fixed in 10% neutral formalin for 48 hr. The tissues were treated with dehydrated ethanol, routinely embedded in paraffin wax and cut into 4 μm sections. Later, the sections were stained with hematoxylin-eosin (HE), and then examined for liver damage under an inverted light microscope at 10 × and 40 × magnification.

***RT-PCR analysis of cytokine expression***

Liver tissues were collected 8 hr after ConA challenge. For RT-PCR analysis, total RNAs were isolated from mouse liver using RNAiso plus regent according to the manufacturer’s protocol. Total RNAs were treated with DNase and protease to remove genomic DNA and protein contamination, and cDNA was then synthesized from 4 μg of total RNA. Quantitative RT-PCR was performed in a 25 μl of reaction solution containing cDNA, primers and 2× EasyTaq PCR SuperMix. The mRNA levels of the pro-inflammatory cytokines IFN-γ, tumor necrosis factor alpha (TNF-α), and IL-17A were assessed by RT-PCR. Primer sequences (Generay Biotech, Shanghai, China) for each target gene are displayed in Table 1. The resulting PCR products for each target were analyzed in 1.2% agarose gels and stained with GelStain (Transgen, Beijing, China).

***Measurement of serum pro-inflammatory cytokines***

Serum samples were collected from mice euthanized at 8 hr after ConA treatment, and the concentrations of the inflammatory cytokines TNF-α, IFN-γ, and IL-17A in the serum were determined by enzyme-linked immunosorbent assays (ELISA) kit (ExCell Bio, Shanghai, China) according to the manufacturer’s instruction.

***Statistical analysis***

Data are reported as the mean±SEM. Differences between groups were evaluated by one-way ANOVA and Bonferroni statistical test. For each analysis, differences were considered statistically significant for *P*<0.05.

## Results

***Exogenous IL-36Ra expression in mice***

**Figure 1.**
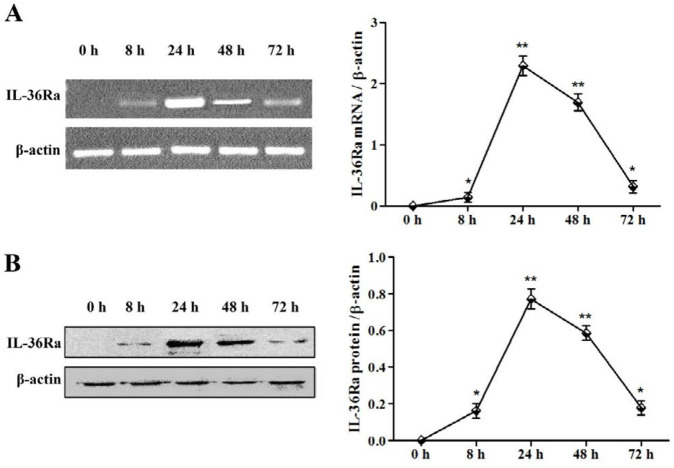
Detection of exogenous interleukin-36 receptor antagonist expression. (A) Reverse transcription-polymerase chain reaction (RT-PCR) analysis of IL-36Ra mRNA levels in the liver. Representative RT-PCR results from animals 0 hr, 8 hr, 24 hr, 48 hr, and 72 hr after hydrodynamic procedures. (B) Western blot analysis of IL-36Ra protein expression in the liver. The left panel shows representative results of mice examined at each time point, while the right panel shows the mean value of all mice examined at each indicated time point. β-actin was used for normalization. (* *P*<0.05, ** *P*<0.01)

Mice were euthanized at 8, 24, 48, and 72 hr, respectively after hydrodynamic-based gene delivery. Liver tissues were acquired for RNA extraction and western blot. IL-36Ra mRNA was checked in the liver by semi-quantitative RT-PCR. RT-PCR detected a unique DNA fragment band of 468 bp corresponding to the expected size of IL-36Ra (Figure 1A). IL-36Ra mRNA was present as early as 8 hr after gene delivery. The highest level was observed 24 hr after gene transfer, and IL-36Ra mRNA then showed a steady time-dependent decrease. However, the IL-36Ra mRNA was still detectable even after 72 hr of gene transfer. 

To confirm the mRNA results, we then checked IL-36Ra protein expression in the liver using western blot. Western blot detected a unique band of approximately 17 kDa corresponding to the molecular weight of IL-36Ra (Figure 1B). Importantly, the levels for IL-36Ra protein in the liver showed a similar trend to the mRNA levels measured previously. The highest protein levels were found at 24 hr of gene transfer. 

***IL-36Ra attenuates ConA-induced liver injury***

**Figure 2:**
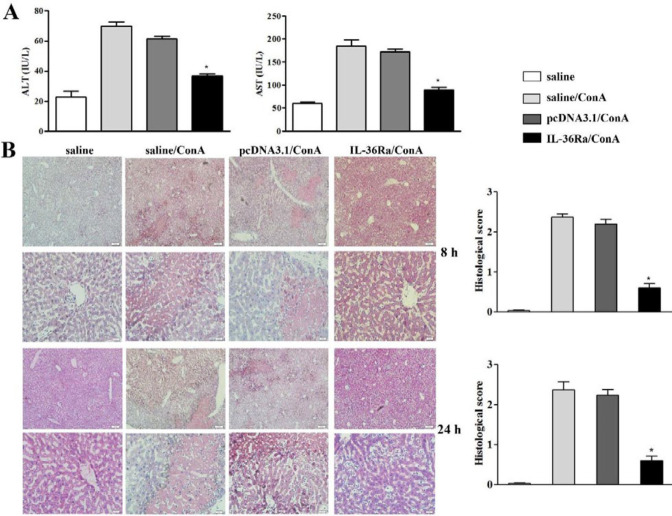
Interleukin-36 receptor antagonist attenuates concanavalin A (ConA)-induced liver injury. (A) Mice with IL-36Ra expression showed significantly lower serum alanine aminotransferase (ALT) and aspartate aminotransferase (AST) levels than that of the ConA control groups (saline/ConA and pcDNA3.1/ConA, * *P*<0.05). (B) Livers obtained 8 hr and 24 hr after ConA injection were subjected to histological analysis of hepatic necrosis using H&E staining. Compared to the normal control mice (saline only), mice treated with ConA (saline/ConA, pcDNA3.1/ConA and IL-36Ra/ConA) displayed varying degrees of liver damage, and large necrotic areas were visible in ConA-treated group mice (saline/ConA and pcDNA3.1/ConA). It was noted that mice administered with the IL-36Ra plasmid had a less severe liver necrosis compared to the ConA alone group mice (saline/ConA, pcDNA3.1/ConA)

The mice in the control groups (saline/ConA and pcDNA3.1/ConA) had a significantly increased serum concentration of ALT and AST after ConA injection. However, the serum ALT and AST levels in mice treated with the IL-36Ra plasmid (IL-36Ra/ConA) were significantly lower than mice of the control group (*P *<0.01) (Figure 2A). These results suggest that IL-36Ra markedly inhibited release of ALT and AST into the plasma of ConA-treated mice. 

The pathological changes in the liver were examined by H&E staining of liver sections at 8 and 24 hr after ConA injection. Massive hepatocyte necrosis in both control groups (pcDNA3.1/ConA and saline/ConA) was observed. In sharp contrast, mice treated with IL-36Ra plasmid (IL-36Ra/ConA) displayed a markedly diminished area and extent of necrosis compared to the control groups (Figure 2B). These pathological changes were consistent with changes in aminotransaminase level. Our results suggest that expression of exogenous IL-36Ra in the liver has a protective effect on ConA-induced hepatitis.

***IL-36Ra suppressed serum pro-inflammatory cytokine production***

**Figure 3:**
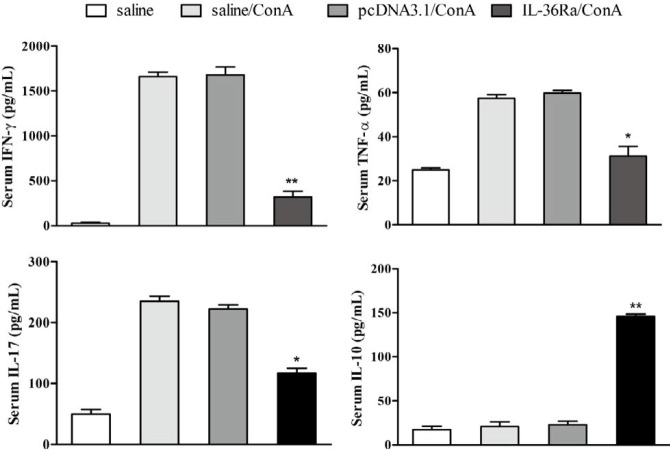
Impact of interleukin-36 receptor antagonist on the serum levels of pro-inflammatory cytokines in concanavalin A (ConA)-induced liver injury. Blood samples were collected by cardiac puncture for the detection of interferon gamma (IFN-γ), tumor necrosis factor alpha (TNF-α), and interleukin-17A (IL-17A) 8 hr after ConA injection. The levels of IFN-γ, TNF-α, and IL-17A were significantly lower in mice administered with IL-36Ra than those in the ConA control groups (saline/ConA and pcDNA3.1/ConA) (*P<0.05, ** *P*<0.01)

As shown in Figure 3, IFN-γ, TNF-α, and IL-17A levels were greatly increased in the ConA-treated control groups (saline/ConA and pcDNA3.1/ConA). Notably, IL-36Ra treatment significantly suppressed ConA-induced production of IFN-γ, TNF-α, and IL-17A in the serum compared to the ConA groups (saline/ConA and pcDNA3.1/ConA).

***IL-36Ra inhibited pro-inflammatory cytokines mRNA expression***

**Figure 4:**
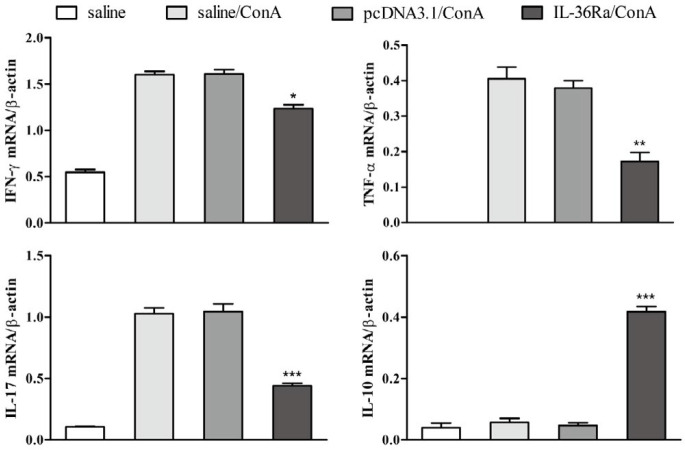
Reverse transcription-polymerase chain reaction analysis of cytokines expression in the liver. Liver tissues were harvested from mice sacrificed 8 hr after ConA injection, and hepatic interferon gamma (IFN-γ), tumor necrosis factor alpha (TNF-α), and interleukin-17A (IL-17A) mRNA levels were analyzed by RT-PCR. β-actin was used as an internal control (*P<0.05, ** *P*< 0.01, *** *P*<0.001)

ConA-induced hepatic injury was associated with changes in the expression of pro-inflammatory cytokines. To further confirm the above observations, we examined mRNA levels of cytokines in the mouse liver at 8 hr after ConA injection. We found that the expression of IFN-γ, TNF-α, and IL-17A mRNA in the liver was significantly increased in the ConA control groups. Importantly, at the same time, the expression of these cytokines in the IL-36Ra-treated group was significantly reduced relative to the ConA groups (Figure 4). 

## Discussion

In the present study, we demonstrated that IL-36Ra was able to protect mice against ConA-induced hepatitis. The serum elevation of liver enzymes was significantly inhibited and liver necrosis was attenuated by IL-36Ra treatment. At the same time, IL-36Ra treatment significantly down-regulated the expression of IFN-γ, TNF-α, and IL-17A. These findings suggest that IL-36Ra is beneficial for protection against acute liver injury induced by ConA. 

IL-36Ra is a new member of the IL-1 family. It is an anti-inflammatory cytokine together with IL-1Ra, IL-37 and IL-38, while the other seven members of IL-1 family are pro-inflammatory cytokines. Previous reports have shown that IL-1Ra (22), IL-37 (23) and IL-38 (24) play a protective role in ConA-induced liver injury. IL-36Ra has been identified as an inhibitory molecule for blocking the activations of NF-κB and MAPKs (11, 12). To address whether IL-36Ra could be a therapeutic target for autoimmune hepatitis, we set out to investigate the significance of IL-36Ra in hepatic injury induced by ConA. The eukaryotic expression plasmid was introduced into mice by the hydrodynamic-based gene delivery procedure. Hydrodynamic injection is by far the most effective method for gene transfer into mouse liver. Another feature of this injection method is that it can transfer a large amount of plasmid solution into mice in 5–8 sec (25). In our experiment, the highest expression of IL-36Ra mRNA and protein was detected at 24 hr after IL-36Ra gene transfer, and there were no deaths caused by the procedure. In this study, IL-36Ra was thus successfully introduced into mice by hydrodynamic-based gene delivery procedure.

Serum ALT and AST levels and histopathology injury are widely used as conventional measurements for the evaluation of hepatic injury (26). Liver injury is usually accompanied by elevated serum ALT and AST levels, and aggravated liver histopathological damage. In this study, we measured liver injury according to the detection of serum concentrations of ALT and AST, and histopathological observations of liver injury using light microscopy. Our results showed that IL-36Ra delivery significantly inhibited liver necrosis, as judged by reduced serum ALT and AST concentrations and histopathological changes after ConA challenge. 

ConA-induced hepatitis is a valid model of immunological hepatic injury. Experimental studies in animals have demonstrated that liver injury in ConA-induced hepatitis is associated with activated CD4^+^T cells, macrophages, and natural killer T (NKT) cells that cause hepatocyte injury by producing lots of pro-inflammatory cytokines, particularly IFN-γ and TNF-α (15, 21, 26-28). Notably, antibodies against IFN-γ and TNF-α can attenuate ConA-induced liver injury in mice (29, 30). IFN-γ is produced by immune cells such as T cells, NK cells, and NKT cells. Aberrant IFN-γ expression has been associated with numerous inflammatory and autoimmune diseases, including autoimmune hepatitis (30). The inflammatory pathway regulated by IFN-γ in the liver also plays a crucial role in immune responses in autoimmune hepatitis (31). IL-17A has been demonstrated to be essential for the development of various autoimmune diseases (32). It is mainly secreted by CD4^+^ T cells, NKT cells, and gamma delta T (γδT) cells, and the liver contains all these cell types (33). Previous studies have shown that IL-17A may exacerbate liver injury by recruiting neutrophils (34, 35). Vigne *et al.* (36, 37) showed that IL-36β promotes IFN-γ production by CD4^+^ T cells and cultured splenocytes, and these stimulatory effects were antagonized by IL-36Ra. Similarly, Gresnigt *et al.* (38) found that IL-36Ra reduces aspergillus-induced IL-17 and IFN-γ production. Consistent with those published data, our experiments showed that IL-36Ra treatment significantly reduced TNF-α, IFN-γ and IL-17A expression in ConA-induced liver injury in mice. 

**Table 1:** Primer sequences used in this study for analysis of pro-inflammatory cytokines

Target gene	Forward primers (5´-3´)	Reverse primers (5´-3´)
β-actin	TCCTGTGGCATCCATGAAACT	GAAGCACTTGCGGTGCACGAT
IFN-γ	TGAACGCTACACACTGCATCTTGG	CGACTCCTTTTCCGCTTCCTGAG
TNF-α	TCTTCTCATTCCTGCTTGTGG	CACTTGGTGGTTTGCTACGAC
IL-17A	TATCCCTCTGTGATCTGGGAAG	ATCTTCTCGACCCTGAAAGTGA

## Conclusion

Our present study revealed that IL-36Ra treatment significantly attenuated ConA-induced liver injury. Hepatic necrosis was significantly inhibited in mice, and serum levels of ALT and AST were reduced accordingly. The protective role of IL-36Ra was associated with the reduced expression of the pro-inflammatory cytokines TNF-α, IFN-γ, and IL-17A. Our results suggest that IL-36Ra may serve as a novel drug target for the treatment of hepatitis.
